# Ultrasonographic evaluation of diaphragm fatigue in healthy humans

**DOI:** 10.1113/EP092322

**Published:** 2025-01-09

**Authors:** Camilla R. Illidi, Lee M. Romer

**Affiliations:** ^1^ Division of Sport, Health and Exercise Sciences, College of Health, Medicine and Life Sciences Brunel University London Uxbridge UK; ^2^ Clinical Exercise and Respiratory Physiology Laboratory, Department of Kinesiology and Physical Education McGill University Montréal Québec Canada

**Keywords:** diaphragm, fatigue, ultrasound

## Abstract

Assessment of diaphragm function and fatigue typically relies on the measurement of transdiaphragmatic pressure (*P*_di_). Although *P*_di_ serves as an index of diaphragm force output, it provides limited information regarding the ability of the muscle to shorten and generate power. We asked whether ultrasonography, combined with *P*_di_, could be used to quantify changes in diaphragm function attributable to fatigue. Eight healthy men [mean (SD) age, 23 (7) years] completed two tasks on separate occasions: (i) 2 min of maximal isocapnic ventilation (MIV); or (ii) 3 × 5 min of maximal inspiratory resistive loading (IRL). Diaphragm function was evaluated before (PRE) and after each task (POST_1_, 10–15 min and POST_2_, 30–35 min) using synchronous recordings of *P*_di_ and subcostal ultrasound traces of the right crural hemidiaphragm during anterolateral magnetic stimulation of the phrenic nerves and progressive CO_2_ rebreathing. Fatigue was quantified as pre‐ to post‐loading changes in twitch *P*_di_, excursion velocity (excursion/time) and power (*P*_di_ × velocity). Both tasks resulted in significant reductions in twitch *P*_di_ (*P *< 0.05). There were no effects of MIV on ultrasound‐derived measures. In contrast, IRL elicited a significant reduction in twitch excursion at POST_1_ (−16%; *P *= 0.034) and significant reductions in excursion velocity at POST_1_ (−32%; *P *= 0.022) and POST_2_ (−28%; *P *= 0.013). These reductions in excursion velocity, alongside the concurrent reductions in twitch *P*_di_, resulted in significant reductions in diaphragm power at POST_1_ (−48%; *P *= 0.009) and POST_2_ (−42%; *P *= 0.008). Neither task significantly altered the contractile responses to CO_2_. In conclusion, subcostal ultrasonography coupled with phrenic nerve stimulation is a promising method for quantifying contractile fatigue of the human diaphragm.

## Abstract (highlights)

- **What is the central question of this study?**
  Can ultrasonography be used to quantify contractile fatigue of the human diaphragm?
- **What is the main finding and its importance?**
  Synchronous recording of ultrasound traces and pressure data during phrenic nerve stimulation proved to be a feasible and effective method for quantifying changes in diaphragm contractile function attributable to fatigue. This approach has the potential to advance our understanding of diaphragm function and fatigue and could be used to inform the development of targeted interventions aimed at improving the contractile properties of the human diaphragm in health and disease.

## 1 INTRODUCTION

The diaphragm is the primary muscle of inspiration in humans. Beyond its critical role in ventilating the lungs, the diaphragm is responsible for venous return of blood to the heart, the maintenance of stable posture and the support of expulsive activities. The standard method for assessment of diaphragm function involves measuring transdiaphragmatic pressure (*P*_di_) in response to electrical or magnetic stimulation of the motor (phrenic) nerves (Laveneziana et al., 2019). Application of these techniques to humans has shown that, like other skeletal muscles, the diaphragm is susceptible to fatigue following repeated or sustained contractions. For instance, external loading of the respiratory muscles via velocity (flow) or force (pressure) tasks has been shown to induce significant reductions in the *P*_di_ response to tetanic or twitch contractions (Romer & Polkey, 2008). A major limitation, however, is that stimulation‐evoked *P*_di_ is a mere index of force output, which offers limited insight into the ability of the muscle to shorten and generate power. This distinction is important because small reductions in force output and shortening velocity can lead to substantial reductions in power output (power = force × velocity).

Animal models have been used to gain a better understanding of the contractile function of the fatigued diaphragm. Studies using these models have shown that static and dynamic contractions can significantly reduce the force output and/or shortening velocity of the muscle (Ameredes & Clanton, 1990; Ameredes et al., 2000; Coirault et al., 1995; Mardini & McCarter, 1987; Road et al., 1987; Seow & Stephens, 1988). However, interpreting the various findings is difficult owing to differences in contraction protocols and measurement procedures. In humans, attempts to quantify fatigue‐induced changes in diaphragm contractile function have typically relied on measurements derived from maximal inspiratory manoeuvres (McCool et al., 1992; Sarmento et al., 2021; Wanderley e Lima et al., 2022). Such manoeuvres are volitional and hence influenced by participant effort and motivation. These factors can vary widely within the same individual over time, potentially leading to inconsistencies and inaccuracies in fatigue assessment. Thus, there is a need for more objective methods for accurate assessment of diaphragm contractile function and its changes owing to fatigue.

Ultrasonography is a non‐invasive, real‐time technique that can be used to assess the anatomical and functional components of the diaphragm (Laursen et al., 2021). Using an anterior subcostal approach, it is possible to measure the amplitude of crural excursion and to calculate its velocity (excursion/time). Although diaphragm ultrasonography is typically used in the intensive care unit to identify diaphragm paralysis (Gerscovich et al., 2001) and monitor patient–ventilator interactions (Umbrello et al., 2015), its application in healthy individuals also holds promise. For example, subcostal ultrasonography, when coupled with respiratory manometry, could be used to provide an index of diaphragm power output (*P*_di_ × velocity). Using this approach, we have shown that diaphragm power during dynamic lower‐limb exercise is preserved via coordinated adjustments in contractile shortening (Illidi & Romer, 2022). To date, only one study has used ultrasonography to assess changes in diaphragm contractile function attributable to fatigue. Kocis et al. (1997), using a piglet model of diaphragm fatigue, observed a decline in the *P*_di_ response to CO_2_‐induced increases in tidal breathing after fatigue was induced via phrenic nerve pacing. Notably, the decline in *P*_di_ was accompanied by an even greater decline in ultrasound‐derived excursion velocity. Collectively, these findings suggest that ultrasonography, when used alongside existing techniques, could provide valuable insights into contractile function and fatigue of the human diaphragm.

The aim of this study, therefore, was to evaluate the feasibility of using subcostal ultrasonography to quantify changes in diaphragm contractile function attributable to fatigue in healthy humans. To achieve this aim, we selected two distinct loading tasks commonly used to study respiratory muscle fatigue: (i) maximal isocapnic ventilation (MIV), representing a flow task characterized by high velocities of shortening; and (ii) maximal inspiratory resistive loading (IRL), representing a pressure task involving high force outputs. To quantify fatigue, we simultaneously recorded *P*_di_ and ultrasound traces during phrenic nerve stimulation and progressive CO_2_ rebreathing, before and after each loading task. We hypothesised that subcostal ultrasonography could be used to quantify changes in diaphragm contractile function attributable to fatigue. Specifically, we reasoned that both tasks would result in significant reductions in diaphragm power owing to decreases in force (*P*_di_) and velocity.

## 2 MATERIALS AND METHODS

### 2.1 Ethical approval

The study adhered to the standards outlined in the *Declaration of Helsinki*, except for registration in a database. All protocols and procedures were approved by the Brunel University London Research Ethics Committee (16371‐A‐Jul/2019‐19984‐1). Following written and verbal explanations of all procedures and risks, potential participants provided written informed consent.

### 2.2 Participants

Eight healthy, recreationally active (moderate exercise ≥150 min/week or vigorous exercise ≥75 min/week) non‐smokers volunteered to participate. Two of the participants had taken part in a previous study in our laboratory (Illidi & Romer, 2022) but were otherwise naive to the loading tasks. We recruited only males owing to well‐documented sex differences in diaphragm fatigue (Geary et al., 2019). Exclusion criteria included: age (limited to 18–40 years), body mass index (limited to 18.5–30.0 kg/m^2^), history of cardiorespiratory or neuromuscular disorders, hypersensitivity to local anaesthetics, and nasal congestion or deviated septum.

### 2.3 Experimental overview

**FIGURE 1:**
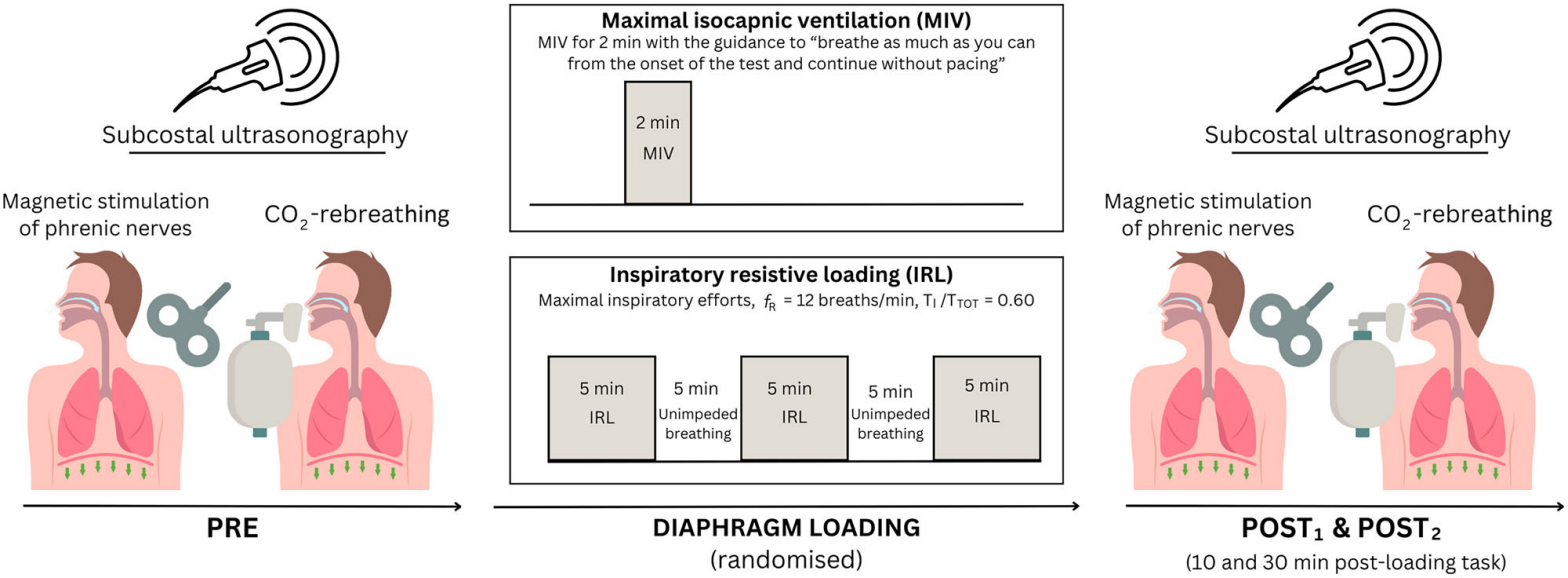
Experimental overview.

The study involved three visits, each separated by 48 h to 7 days. The initial visit was for screening, assessment of baseline characteristics and familiarization with the testing procedures. The subsequent two visits were for the experimental trials, during which the participants completed two respiratory loading tasks aimed at inducing diaphragm fatigue: (i) a low‐force, high‐velocity task (MIV); or (ii) a high‐force, low‐velocity task (maximal IRL). The order of the experimental trials was randomized and counterbalanced, with each trial conducted at the same time of day. To assess diaphragm fatigue, *P*_di_ and ultrasound‐derived responses to phrenic nerve stimulation and progressive CO_2_ rebreathing were evaluated before (PRE) and at two time points after each loading task (POST_1_ and POST_2_). To avoid twitch potentiation (Mador et al., 1994; Wragg et al., 1994), phrenic nerve stimulation was conducted before (after a 10 min rest) and 10 and 30 min after loading, whereas CO_2_ rebreathing was performed immediately before and 15 and 35 min after loading (Figure 1). Participants were instructed to avoid vigorous exercise for ≥24 h, caffeine and alcohol for 12 h, and food for 3 h before all visits.

### 2.4 Visit 1

#### 2.4.1 Participant characteristics and familiarization

Anthropometrics, pulmonary function and diaphragm characteristics (thickness, thickening and excursion) were assessed as described previously (Illidi & Romer, 2022). In addition, participants underwent thorough familiarization with the experimental protocols, including phrenic nerve stimulation, CO_2_ rebreathing and a shortened trial of each loading task (1 min MIV and 5 min IRL).

### 2.5 Visits 2 and 3

#### 2.5.1 Maximal isocapnic ventilation

Participants were seated upright with a nose‐clip in place and breathed on a flanged mouthpiece connected in series to a flow turbine and a directional control valve (112050‐2100, Hans Rudolph, Shawnee, KS, USA). To maintain isocapnia, the mouthpiece–valve assembly was connected to an open‐ended rebreathe tube (i.d. 3 cm, length 60 cm, volume 0.42 L), and humidified gas (5% CO_2_–95% O_2_) was introduced into the distal end of the tube whenever end‐tidal partial pressure of CO_2_ ($P_{\mathrm{ET},CO_{2}}$) dropped below baseline values. Participants were instructed to perform MIV for 2 min with the guidance to ‘breathe as much as you can from the onset of the test and continue without pacing’. Verbal encouragement was provided, including feedback every 30 s about the time remaining. Minute ventilation was displayed on a computer screen, and participants were encouraged to maintain the value as high as possible throughout the test. This loading protocol has been shown to induce significant, long‐lasting reductions in twitch *P*_di_ (Hamnegård et al., 1996; Luo et al., 2001).

#### 2.5.2 Inspiratory resistive loading

While seated upright with a nose‐clip in place, participants breathed on a flanged mouthpiece connected in series to a flow turbine and a customized non‐rebreathing valve (PY2 50‐0975, Harvard Apparatus, Cambridge, UK). Inspiratory resistance was imposed by semi‐occluding the inspiratory port of the valve by means of a rubber stopper with individualized orifice size (i.d. 2.5–4.0 mm); no load was added during expiration. The correct orifice size for each participant was determined during the familiarization visit. The protocol consisted of 3 × 5 min intervals of loaded breathing, with each interval separated by 5 min of normal, unimpeded breathing. Participants were instructed to perform maximal inspiratory efforts at a respiratory frequency (ƒ_R_) of 12 breaths/min and an inspiratory duty cycle [inspiratory time (*t*_I_)/total time (*t*_TOT_)] of 0.60, with assistance from dual‐tone auditory cues. To engage their diaphragm actively, participants placed one hand lightly on their abdomen and emphasized movement of the abdomen during inspiratory efforts. Furthermore, participants were asked to achieve maximal voluntary *P*_di_ and to maintain this pressure as a square wave throughout inspiration by targeting waveforms displayed on a computer screen. Throughout the task, $P_{\mathrm{ET},CO_{2}}$ and earlobe O_2_ saturation were monitored closely. A similar protocol has been shown to elicit significant, long‐lasting reductions in twitch *P*_di_ (Luo et al., 2001).

#### 2.5.3 Phrenic nerve stimulation

**FIGURE 2:**
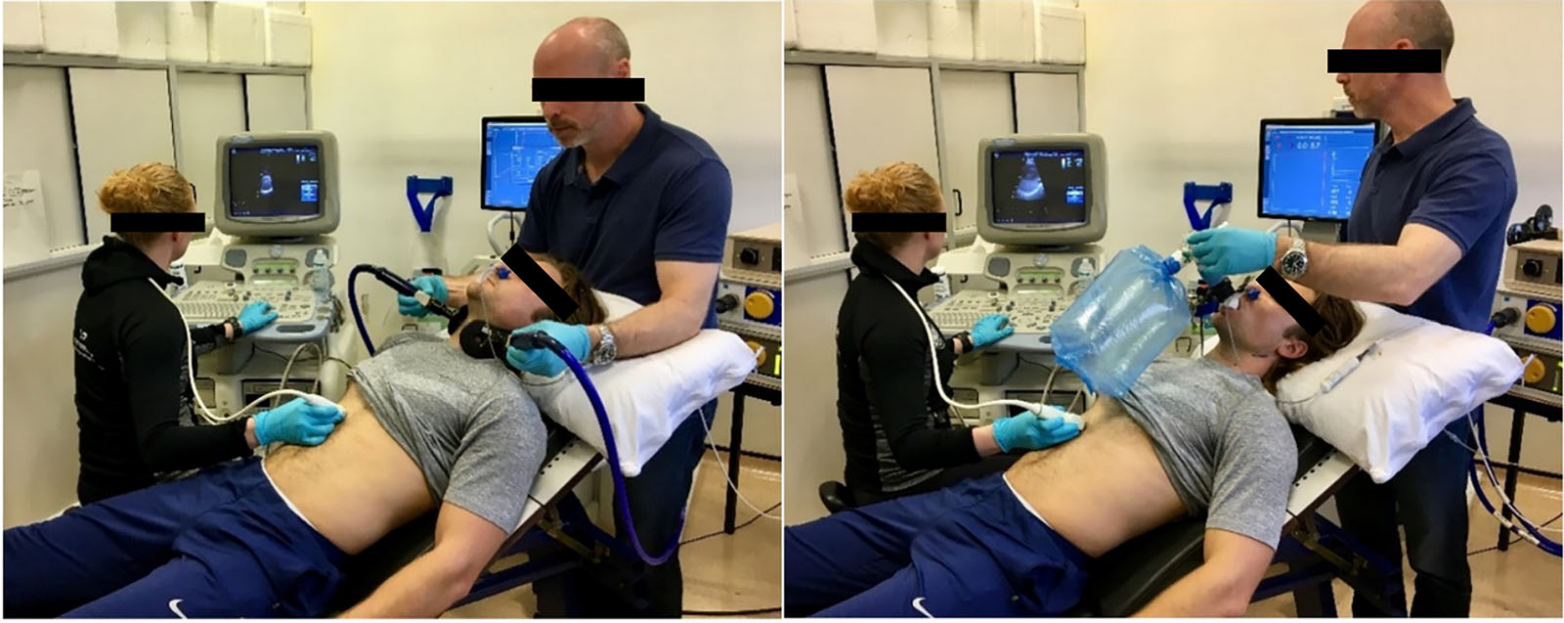
Experimental set‐up for the assessment of diaphragm excursion over time during phrenic nerve stimulation (left) and CO_2_ rebreathing (right).

Phrenic nerve stimulation was conducted while participants adopted a semi‐recumbent position (Figure 2). The phrenic nerves were stimulated using two figure‐of‐eight coils (D70 Alpha B.I., Magstim, Whitland, UK) positioned on the posterior borders of the sternocleidomastoids (Mills et al., 1996). The coils were connected to two magnetic stimulators (Magstim 200), each linked to a BiStim module for simultaneous triggering. Optimal coil positions were identified by moving each coil around the participant's neck while stimulating at 70% intensity until the highest twitch transdiaphragmatic pressure (*P*_di,tw_) was obtained. These positions were marked and kept constant for the duration of the trial. All subsequent stimulations were conducted at functional residual capacity (verified by monitoring oesophageal pressure immediately before stimulation) and against a closed glottis (mouth closed at the end of tidal expiration). To determine whether stimulation intensity was supramaximal, three single twitches were obtained every 30 s at 50%, 60%, 70%, 80%, 85%, 90%, 95% and 100% of maximal stimulator output prior to each task. To assess fatigue, five stimulations at 100% maximal stimulator output were performed at baseline and at 10 and 30 min after each loading task (at POST_1_ and POST_2_, respectively). Diaphragm fatigue was considered present if there was a >10% reduction in the group mean *P*_di,tw_, relative to baseline, after loading. This definition is based on a change in *P*_di,tw_ two to three times greater than the within‐day, between‐occasion coefficient of variation (CV) for this measure (Taylor & Romer, 2009; Taylor et al., 2010; Tiller et al., 2017). All stimulations were separated by 30 s to avoid postactivation potentiation (Mador et al., 1994; Wragg et al., 1994).

#### 2.5.4 CO_2_ rebreathing

The protocol was similar to that described by Read (1967), but with a baseline gas composition of 5% CO_2_–95% O_2_ instead of 7% CO_2_–93% O_2_. Participants adopted the same position as described for phrenic stimulation (Figure 2). With a nose‐clip in place, the participants breathed ambient air for 3 min through a mouthpiece–valve assembly (2110, Hans Rudolph; 54 mL dead space). Next, they exhaled to residual volume, at which point the valve was closed. Participants equilibrated with the rebreathing circuit by taking three deep, rapid breaths from a rebreathing bag filled with the test gas (bag volume ≈ vital capacity + 1 L), followed by the instruction to ‘close your eyes, relax and breathe as needed’. The rebreathing circuit was reopened to ambient air when $P_{\mathrm{ET},CO_{2}}$ reached 55 mmHg or when the participant was unable to tolerate the hypercapnic gas. To minimize the potential influence of external stimuli on ventilatory responses, the laboratory was kept silent during each rebreathing trial. Pulmonary ventilation, gas exchange and respiratory pressures were recorded throughout the test, whereas diaphragm ultrasound traces were recorded at regular intervals.

#### 2.5.5 Pulmonary ventilation and gas exchange

Ventilatory and gas‐exchange indices were assessed breath by breath using an online system consisting of a turbine flowmeter, sample line, and O_2_ and CO_2_ gas analysers (Oxycon Pro, Jaeger, Viasys Healthcare, Hoechberg, Germany). The flowmeter and gas analysers were calibrated before each CO_2_ rebreathing trial and loading task. Digital signals from the online system were captured at 100 Hz using a data acquisition system (Micro1401mk‐II and Spike2, CED, Cambridge, UK) and external device (DAQ‐30A16, Eagle Technology, Cape Town, South Africa) for the precise time alignment of airflow and respiratory pressure data.

#### 2.5.6 Respiratory pressures

Oesophageal and gastric pressures (*P*_oe_ and *P*_ga_, respectively) were measured using a gastro‐oesophageal catheter (Gaeltec Devices, Dunvegan, Isle of Skye, UK), as described previously (Tiller et al., 2018, 2017). Following administration of 2% lidocaine gel to the nasal mucosa, the catheter was passed via the participant's nostril until the pressure transducers showed positive pressure deflections during inspiration. The catheter was then withdrawn until inspiration elicited negative and positive deflections in *P*_oe_ and *P*_ga_, respectively. The catheter was withdrawn an additional 10 cm such that the oesophageal transducer was positioned in the lower third of the oesophagus. Verification was performed using an occlusion test (Baydur et al., 1982). Once the correct position was verified, the catheter was securely taped in position at the nose. The analog signals were amplified (1902, CED), digitized at 200 Hz (1401mk‐II, CED) and recorded (Spike2, CED). Instantaneous *P*_di_ was calculated by online subtraction of *P*_oe_ from *P*_ga_.

#### 2.5.7 Diaphragm shortening

**FIGURE 3:**
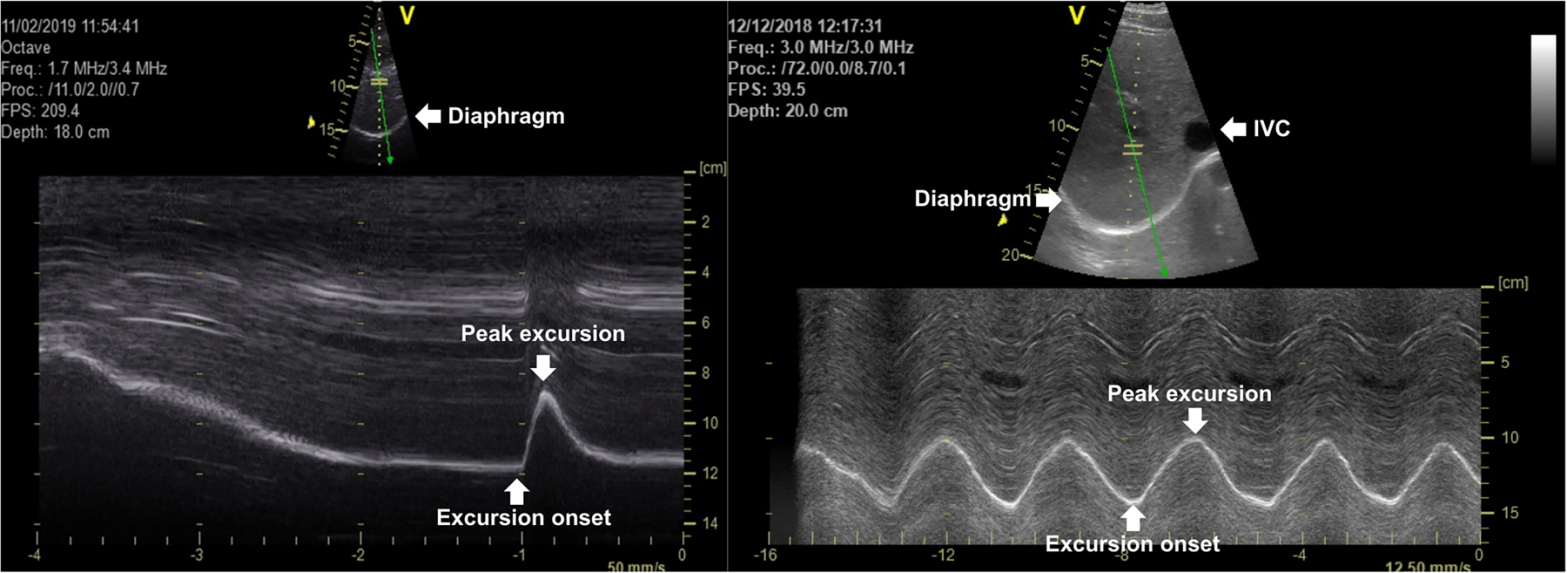
Representative B‐mode (top) and anatomical M‐mode (bottom) ultrasound traces of the right crural hemidiaphragm at baseline (PRE) in response to single ‘twitch’ stimulation of the phrenic nerves at relaxation volume (left panel) and during the final 15 s of CO_2_ rebreathing (end‐tidal partial pressure of CO_2_, 55 mmHg; inspiratory minute ventilation, 32 L/min) (right panel). B‐Mode was used initially to obtain the best diaphragmatic delineation, with the inferior vena cava (IVC) as a landmark to ensure consistency in the positioning of the ultrasound transducer. In anatomical M‐mode, diaphragm excursion (*y*‐axis) and excursion time (*x*‐axis) were measured for the determination of excursion velocity, work and power.

Diaphragm shortening was evaluated using a commercially available ultrasound system (Vivid 7 Pro, GE Medical, Horten, Norway). For phrenic stimulation, a low‐frequency phased‐array transducer (1.5–4.0 MHz; M3S) was positioned subcostally on the right midclavicular line and directed cranially to visualize the posterior portion of the right hemidiaphragm dome (Laursen et al., 2021). Beam penetration depth was adjusted to ensure that the hyperechoic diaphragm at residual volume was always within the field of view (∼200–250 mm). To optimize lateral resolution, one focal point was set at the diaphragm position at relaxation volume. In brightness (B‐)mode, the image width was adjusted to focus specifically on the apex of the hemidiaphragm. This configuration provided an adequate sampling frequency (200–220 frames/s) to capture the rapid twitch response. For CO_2_ rebreathing, a low‐frequency curved‐array transducer (2.4–5.0 MHz; 3.5C) was positioned as described above. The sampling frequency was set to 40–60 frames/s, with a wide field of view from the costophrenic angle laterally to the inferior vena cava medially. Again, penetration depth was adjusted to ensure that the hyperechoic diaphragm was always within the field of view. The sites of transducer placement were marked to ensure consistent replication during subsequent scans. All ultrasound cine loops were acquired in B‐mode, then analysed offline using angle‐independent (anatomical) motion (M‐)mode (EchoPac, v.6.1, GE Medical) (Orde et al., 2016). A digital calliper tool was used to measure diaphragm excursion as the swing amplitude of diaphragm displacement during contraction (Figure 3). Excursion time was determined as the time from the onset of excursion to peak excursion. Mean excursion velocity was calculated as diaphragm excursion/excursion time. Diaphragm work was calculated as force (*P*_di_) × excursion. Diaphragm power was calculated as force (*P*_di_) × velocity (i.e., work/time) (Illidi & Romer, 2022).

### 2.6 Data processing and time matching

Twitch *P*_di_ response to phrenic nerve stimulation was quantified as the change in pressure from stimulus onset to peak pressure. To determine whether muscle fibre lengths and abdominal compliance were uniform across time, end‐expiratory oesophageal and gastric pressures (EEP_oe_ and EEP_ga_, respectively) were measured at the point of stimulation. Ventilatory indices [inspiratory minute ventilation ($\dot{V}$_I_), inspiratory tidal volume (*V*_TI_), ƒ_R_, *t*_I_ and *t*_I_/*t*_TOT_] and respiratory pressures during external loading (MIV and IRL) and CO_2_ rebreathing were recorded breath by breath. The start and end of each breath were marked at points of zero flow, and any anomalous breaths (e.g., those involving swallows, coughs, sighs or failing to cross zero flow) were excluded manually. Ventilatory indices were determined for inspiration using custom scripts. Tidal *P*_di_ was expressed as mean inspiratory pressure ($\bar{P}$_di_). The active component of $\bar{P}$_di_ ($\bar{P}$_di,a_), representing the pressure required for inspiration (i.e., dynamic contraction of the diaphragm), was calculated by subtracting the lowest pressure during any given respiratory cycle from the instantaneous pressure; this index of force output was used to calculate the work and power of the diaphragm during CO_2_ rebreathing (Illidi & Romer, 2022). Diaphragm force output during MIV and IRL was calculated as the time integral of *P*_di_ during inspiration (∫*P*_di_ d*t*) and the cumulative pressure–time product for the entire duration of the task (Σ∫*P*_di_ d*t* × *f*_R_). Diaphragm pressure–time index (PTI_di_) was calculated as the product of the ratio of $\bar{P}$_di_ to maximal *P*_di_ and the inspiratory duty cycle (*t*_I_/*t*_TOT_) (Bellemare & Grassino, 1982). The pressure contribution of ribcage muscles relative to that of the diaphragm during inspiration was calculated as the ratio of *P*_di_ to *P*_oe_. Careful consideration was given to the timing of ultrasound acquisition during CO_2_ rebreathing to ensure accurate identification and matching of inspiratory cycles within each ultrasound cine loop with the breath‐by‐breath ventilatory and pressure data (Illidi & Romer, 2022). Briefly, 15 s ultrasound cine loops were recorded twice at rest and every 30 s during hypercapnic hyperpnoea, with all breaths within each cine loop identified and averaged over 15 s (30 s for resting eupnoea).

### 2.7 Statistics

Statistical analyses were conducted using dedicated software (SPSS v.26, IBM Corp., Armonk, NY, USA). Data normality was assessed by evaluating skewness (cut‐off value ±1) and kurtosis (cut‐off value ±2). Variables not normally distributed were log_10_‐transformed before further analysis. Between‐task differences in ventilatory indices ($\dot{V}$_I_, *V*_TI_, *f*_R_, *t*_I_ and *t*_I_/*t*_TOT_) and inspiratory pressures (∫*P*_di_ d*t*, Σ∫*P*_di_ d*t* × *f*_R_, PTI_di_, $\bar{P}$_di_/*P*_di,max_ and *P*_di_/*P*_oe_) were assessed using Student's two‐tailed paired‐samples *t*‐tests. For group mean data, repeated‐measures ANOVA with planned pairwise comparisons was used to determine whether a plateau in the absolute values for *P*_di,tw_ occurred with increasing stimulation intensity for each loading task. Mauchly's sphericity test was used to check for homogeneity of covariance, and the Greenhouse–Geisser adjustment was applied when the assumption of sphericity was violated. For individual participant data, a plateau in *P*_di,tw_ was considered present if the difference in *P*_di,tw_ between submaximal and maximal stimulation intensities was less than or equal to the within‐block CV for all twitches (Geary et al., 2019). For pressure responses (EEP_oe_, EEP_ga_ and *P*_di,tw_) and ultrasound‐derived responses (excursion, time, velocity, work and power) to phrenic nerve stimulation, between‐task differences at baseline (PRE) and within‐task differences at specific time points (PRE vs. POST_1_ and PRE vs. POST_2_) were analysed using Student's paired‐samples *t*‐tests. For CO_2_ rebreathing, the rates of change (slopes) in ventilatory and pressures indices (*y*‐axis) as a function of $P_{\mathrm{ET},CO_{2}}$ (*x*‐axis) were quantified using least‐squares linear regression and compared using Student's paired‐samples *t*‐tests. The ventilatory, pressure and ultrasound‐derived responses at discrete $P_{\mathrm{ET},CO_{2}}$ levels of 45 and 55 mmHg were subsequently identified using linear interpolation. Between‐task differences at PRE and within‐task differences at each level of $P_{\mathrm{ET},CO_{2}}$ across time points (PRE vs. POST_1_ and PRE vs. POST_2_) were analysed using Student's paired‐samples *t*‐tests. Owing to the exploratory nature of our analyses, we did not adjust for multiple comparisons (Sainani, 2009). However, we did consider the pattern of effect sizes by reporting Cohen's *d_z_* for dependent samples, with the magnitude of the observed effects interpreted as small (*d_z_* = 0.20), medium (*d_z_* = 0.50) or large (*d_z_* = 0.80) (Cohen, 1988). Data are presented as the mean (SD). Statistical significance was set at *P* < 0.05.

## 3 RESULTS

### 3.1 Participant characteristics

Participant characteristics are shown in Table 1. All participants exhibited values for pulmonary function within normal limits. Indices pertaining to diaphragm thickness, thickening and excursion were consistent with values reported previously (Laursen et al., 2021).

**TABLE 1:** Participant characteristics.

Parameter	Measured value	Percentage predicted
Anthropometrics		
Age (years)	23 (7)	–
Stature (m)	1.80 (0.05)	–
Body mass (kg)	77.1 (10.3)	–
Body mass index (kg/m^2^)	23.7 (3.0)	–
Chest circumference (cm)	94.4 (2.6)	–
Chest depth (cm)	20.0 (1.1)	–
Chest width (cm)	30.3 (1.7)	–
Pulmonary function		
TLC (L)	7.46 (0.87)	107 (10)
RV (L)	1.86 (0.23)	112 (14)
FRC_pleth_ (L)	3.97 (0.72)	116 (18)
FVC (L)	5.74 (0.47)	100 (5)
FEV_1_ (L)	4.75 (0.41)	101 (7)
FEV_1_/FVC	0.82 (0.05)	100 (4)
MVV_12_ (L/min)	197 (28)	108 (14)
PI_max_ (cmH_2_O)	−125 (16)	113 (15)
PE_max_ (cmH_2_O)	173 (19)	112 (11)
Diaphragm characteristics		
Thickness at FRC (mm)	1.6 (0.5)	–
Thickness at TLC (mm)	4.1 (0.7)	–
Thickening fraction	1.56 (0.40)	–
Maximal excursion (cm)	6.78 (1.50)	–

*Notes*: Data are means (SD) for eight participants. Predicted values were derived from Quanjer et al. (2012) for spirometry, Stocks and Quanjer (1995) for plethysmography, and Evans and Whitelaw (2009) for manometry. Predicted values for MVV_12_ were calculated as FEV_1_ × 40.

Abbreviations: FEV_1_, forced expiratory volume in 1 s; FRC_pleth_, plethysmography‐derived functional residual capacity; FVC, forced vital capacity; MVV_12_, maximal voluntary ventilation in 12 s; PE_max_, maximal expiratory mouth pressure from TLC; PI_max_, maximal inspiratory mouth pressure from RV; RV, residual volume; TLC, total lung capacity.

### 3.2 Respiratory loading

Quantitative characteristics of the two loading tasks are shown in Table 2. No differences were noted across the three intervals of IRL; therefore, the data are presented for all three intervals combined. For MIV, breath‐by‐breath inspiratory ventilation ($\dot{V}$_I_) peaked at 9 (5) s [216 (22) L/min], then declined rapidly until a plateau phase was reached at 55 (13) s. At the plateau, $\dot{V}$_I_ was 72 (5)% of the initial peak. By design, diaphragm force output (∫P_di_ d*t*) was lower and mean inspiratory flow (*V*_TI_/T_I_) higher during MIV compared with IRL (both *P *< 0.001), indicating significantly different loading profiles (i.e., MIV: low force, high velocity vs. IRL: high force, low velocity). The cumulative force output (Σ∫*P*_di_ d*t* × *f*_R_) and the pressure–time index of the diaphragm (PTI_di_) were also lower during MIV (both *P *< 0.001).

**TABLE 2:** Mean ventilatory and pressure responses to maximal isocapnic ventilation and inspiratory resistive loading.

Parameter	MIV	IRL
$\dot{V}$_I_ (L/min)	170 (7)	22 (6)^*^
*V*_TI_ (L)	1.97 (0.37)	1.98 (0.55)
*f*_R_ (breaths/min)	86.6 (19.0)	11.0 (0.8)^*^
*V*_TI_/*t*_I_ (L/s)	5.95 (0.63)	0.75 (0.20)^*^
*t*_I_/*t*_TOT_	0.47 (0.01)	0.56 (0.06)^*^
∫*P*_di_ d*t* (cmH_2_O × s)	20.9 (6.9)	56.9 (13.1)^*^
Σ∫*P*_di_ d*t* × *f*_R_ (cmH_2_O × s/min)	3566 (1093)	9467 (2416)^*^
PTI_di_	0.16 (0.07)	0.43 (0.06)^*^
$\bar{P}$_di_/*P*_di,max_	0.33 (0.16)	0.77 (0.05)^*^
$\bar{P}$_di_/$\bar{P}$_oe_	2.28 (1.17)	1.47 (0.39)^*^

*Note*: Data are means (SD) for eight participants.

Abbreviations: *f*_R,_ respiratory frequency; IRL, inspiratory resistive loading; MIV, maximal isocapnic ventilation; $\bar{P}$_di_/*P*_di,max_, mean inspiratory transdiaphragmatic pressure relative to maximal transdiaphragmatic pressure; $\bar{P}$_di_/$\bar{P}$_oe_, mean inspiratory transdiaphragmatic pressure relative to mean inspiratory oesophageal pressure; PTI_di_, diaphragm pressure–time index; *t*_I_/*t*_TOT_, inspiratory duty cycle; $\dot{V}$_I_, inspiratory minute ventilation; *V*_TI_, inspiratory tidal volume; *V*_TI_/*t*_I_, mean inspiratory flow; Σ∫*P*_di_ d*t*, sum of time integral of transdiaphragmatic pressure during inspiration over the duration of the loading task (i.e., cumulative diaphragm force output); ∫*P*_di_ d*t*, time integral of transdiaphragmatic pressure during inspiration (i.e., diaphragm force output per breath).

* *P* < 0.05 versus MIV.

### 3.3 Phrenic nerve stimulation

**FIGURE 4:**
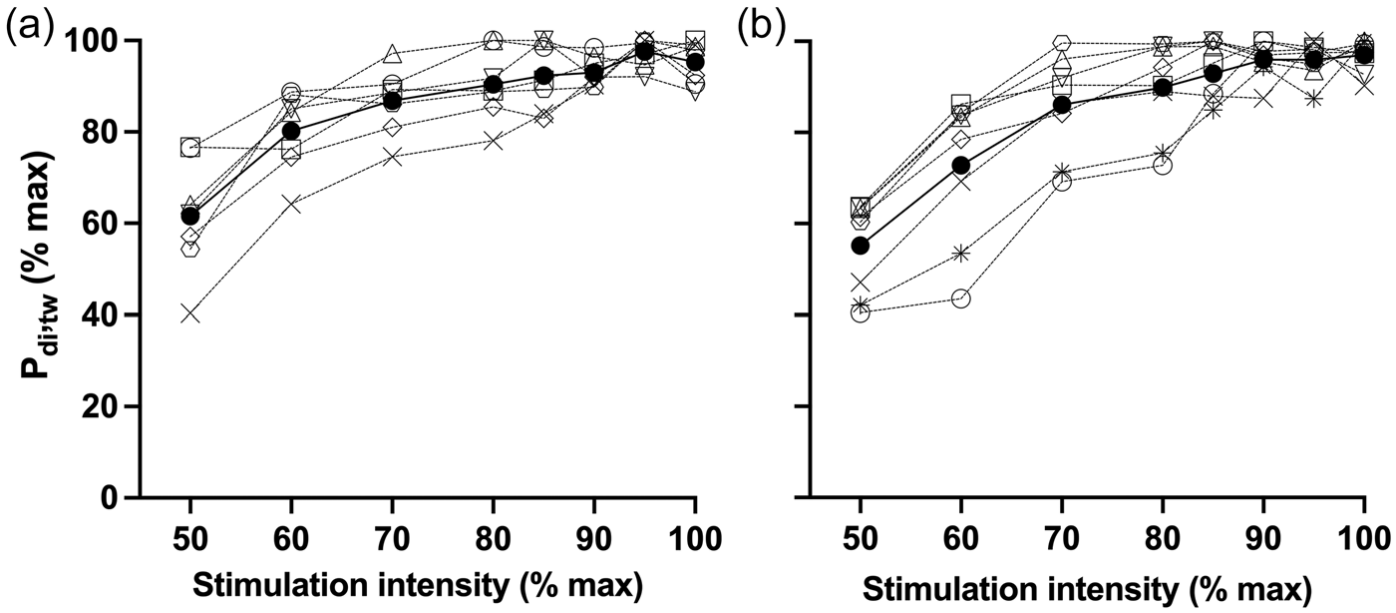
Individual participant (open symbols and dotted lines) and group mean (filled circles and continuous line) *P*_di,tw_ in response to phrenic nerve stimulation of increasing intensity prior to MIV (a) and IRL (b). Data are for eight participants. Abbreviations: IRL, inspiratory resistive loading; MIV, maximal isocapnic ventilation; *P*_di,tw_, twitch transdiaphragmatic pressure.

Plateaus in *P*_di,tw_ occurred with increasing stimulation intensity (Figure 4), thereby confirming supramaximal stimulation for both tasks. For group mean data, there were no consecutive differences in *P*_di,tw_ at stimulation intensities ≥80% of maximum (*P* > 0.05). For individual data, all participants exhibited a plateau between 80% and 90% of maximum.

**FIGURE 5:**
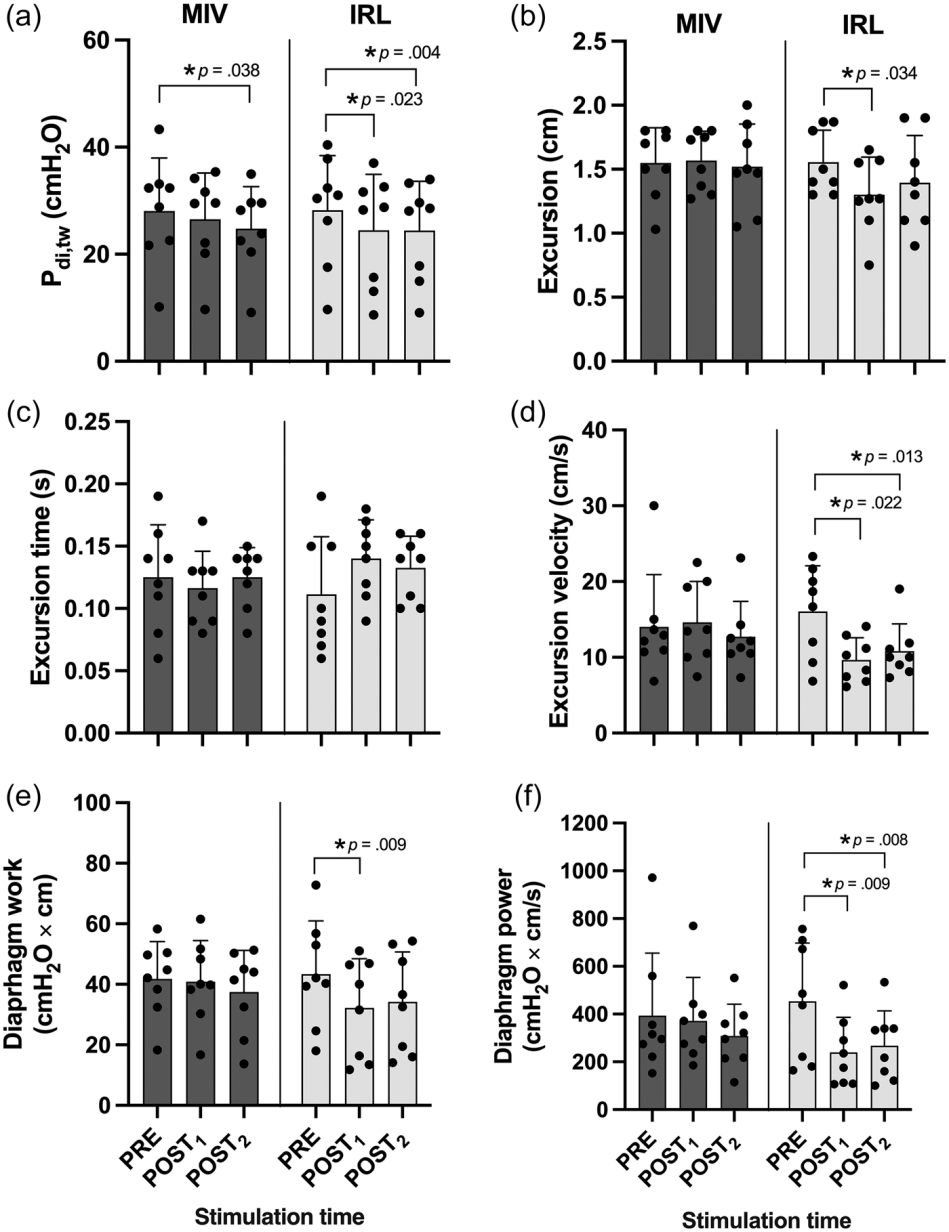
Diaphragm pressure and shortening responses to phrenic nerve stimulation at baseline and at two time points after MIV and IRL. Individual data points are shown for eight participants. Bars and whiskers represent the mean (SD), respectively. ^*^ *P* < 0.05 versus PRE. Abbreviations: MIV, maximal isocapnic ventilation; IRL, inspiratory resistive loading; *P*_di,tw_, twitch transdiaphragmatic pressure.

Pressure and ultrasound data are shown in Figure 5. There were no significant effects of task or time on EEP_oe_ or EEP_ga_ (all *P* > 0.05; data not shown). For MIV, there was a 5% reduction in *P*_di,tw_ at POST_1_ (*P* = 0.206; *d_z_ *= 0.165) and a 12% reduction at POST_2_ (*P* = 0.038; *d_z_ *= 0.915). For IRL, the reductions were more pronounced: 14% at POST_1_ (*P* = 0.023; *d_z_ *= 1.021) and 13% at POST_2_ (*P* = 0.004; *d_z_ *= 1.463). Irrespective of the post‐task time point, a reduction in *P*_di,tw_ of >10% was noted in three participants after MIV and five after IRL.

Clear and stable ultrasound traces were observed for all stimulations (see also Figure 3). No between‐task differences were noted at PRE. Ultrasound‐derived measures were unaffected by MIV. In contrast, IRL resulted in a significant reduction in diaphragm excursion at POST_1_ (−16%; *P* = 0.034, *d_z_* = 1.170), with only partial recovery by POST_2_ (−9%; *P* = 0.196, *d_z_* = 0.600). These reductions in excursion were accompanied by increases in excursion time at POST_1_ (44%; *P* = 0.132, *d_z_* = 0.880) and POST_2_ (32%; *P* = 0.147, *d_z_* = 0.639), resulting in significant reductions in excursion velocity at POST_1_ (−32%; *P* = 0.022, *d_z_* = 1.490) and POST_2_ (−28%; *P* = 0.013, *d_z_* = 1.457). The effect of IRL on both *P*_di,tw_ and diaphragm excursion was a significant reduction in diaphragm work at POST_1_ (−27%; *P* = 0.009, *d_z_* = 0.849) and a return towards baseline at POST_2_ (−20%; *P* = 0.077, *d_z_* = 0.691). Likewise, the combined effect of IRL on *P*_di,tw_ and excursion velocity was a significant reduction in diaphragm power output at POST_1_ (−48%; *P* = 0.009, *d*_z_ = 1.232) and POST_2_ (−42%; *P* = 0.008, *d_z_* = 1.188).

### 3.4 CO_2_ rebreathing

All participants tolerated the hypercapnic–hyperoxic gas mixture. Ventilatory and breathing pattern responses are presented in Table 3. In all participants, the $\dot{V}$_I_ versus $P_{\mathrm{ET},CO_{2}}$ slopes (pooled data) were linear with high correlation coefficients for both MIV [*r* = 0.70 (0.25); SEE = 3.79 (1.60) L/min] and IRL [*r* = 0.75 (0.18), SEE = 1.60 (1.26) L/min]. At PRE, the individual participant $\dot{V}$_I_ versus $P_{\mathrm{ET},CO_{2}}$ slopes ranged from 0.28 to 5.50 L/min/mmHg, and the group mean slopes were similar for MIV and IRL [1.47 (0.71) vs. 1.32 (1.06) L/min/mmHg, *P* = 0.617]. Furthermore, the $\dot{V}$_I_ versus $P_{\mathrm{ET},CO_{2}}$ slopes remained stable over time for both tasks. The ventilatory responses during quiet breathing (eupnoea) were similar for MIV and IRL. From eupnoea to 55 mmHg $P_{\mathrm{ET},CO_{2}}$ (pooled data), $\dot{V}$_I_ increased by a factor of 2.5. Owing to negligible changes in breath timing, *V*_TI_/T_I_ and *V*_TI_ increased to similar extents (2.5‐ and 2.2‐fold, respectively).

**TABLE 3:** Ventilatory and breathing pattern responses at two levels of end‐tidal partial pressure of CO_2_ at baseline and at two time points after maximal isocapnic ventilation and inspiratory resistive loading.

	PRE		POST_1_		POST_2_	
Parameter	45 mmHg	55 mmHg	45 mmHg	55 mmHg	45 mmHg	55 mmHg
MIV						
$\dot{V}$_I_ (L/min)	16.8 (9.6)	31.5 (8.8)	14.9 (7.9)	29.9 (9.8)	13.0 (5.8)	26.0 (7.0)
*V*_TI_ (L)	1.29 (0.75)	2.11 (0.65)	1.07 (0.79)	2.01 (0.60)	1.34 (0.74)	2.11 (0.66)
*f*_R_ (breaths/min)	14.2 (4.8)	15.1 (3.6)	12.0 (4.8)	14.7 (2.5)	10.8 (4.9)	14.7 (3.3)
*t*_I_ (s)	2.16 (0.91)	2.23 (1.13)	2.02 (0.60)	1.91 (0.31)	2.49 (0.90)	1.81 (0.42)
*t*_I_/*t*_TOT_	0.45 (0.04)	0.47 (0.04)	0.43 (0.06)	0.47 (0.05)	0.47 (0.05)	0.46 (0.07)
*V*_TI_/*t*_I_ (L/s)	0.59 (0.20)	1.05 (0.42)	0.52 (0.26)	1.05 (0.32)	0.53 (0.20)	1.16 (0.57)
IRL						
$\dot{V}$_I_ (L/min)	10.1 (5.3)	23.4 (11.1)	12.7 (5.9)	31.3 (20.4)	12.9 (7.7)	30.4 (15.8)
*V*_TI_ (L)	0.98 (0.60)	1.96 (0.78)	1.08 (0.67)	2.09 (0.91)	1.23 (0.66)	2.15 (0.68)
*f*_R_ (breaths/min)	11.7 (4.3)	13.1 (3.5)	11.9 (5.9)	15.1 (5.8)	10.9 (5.2)	13.9 (4.5)
*t*_I_ (s)	2.15 (0.76)	2.30 (0.82)	2.25 (0.82)	2.14 (1.19)	2.11 (0.77)	2.08 (0.83)
*t*_I_/*t*_TOT_	0.46 (0.04)	0.47 (0.08)	0.45 (0.05)	0.44 (0.06)	0.44 (0.06)	0.43 (0.08)
*V*_TI_/*t*_I_ (L/s)	0.45 (0.21)	0.85 (0.50)	0.48 (0.28)	0.97 (0.53)	0.58 (0.18)	1.03 (0.56)

*Note*: Data are means (SD) for eight participants.

Abbreviations: *f*_R_, respiratory frequency; IRL, inspiratory resistive loading; MIV, maximal isocapnic ventilation; *t*_I_, inspiratory time; *t*_I_/*t*_TOT_, inspiratory duty cycle; $\dot{V}$_I_, inspiratory minute ventilation; *V*_TI_, inspiratory tidal volume; *V*_TI_/*t*_I_, mean inspiratory flow.

^*^ *P* < 0.05 versus PRE at same end‐tidal partial pressure of CO_2_.

Clear and stable ultrasound traces were observed at all levels of ventilation (Figure 3). Mean (SD) values for pressure and ultrasound data are shown in Table 4. There were no effects of task or time on resting measurements during eupnoeic breathing. Except for excursion time, which remained relatively stable, all other measures increased in line with the hypercapnic stimulus. From eupnoea to 55 mmHg $P_{\mathrm{ET},CO_{2}}$ (pooled data at PRE), pressure and ultrasound measures increased by a factor of 1.9 ($\bar{P}$_di,a_), 1.9 (excursion), 1.9 (velocity), 2.7 (work) and 3.3 (power). At discrete levels of $P_{\mathrm{ET},CO_{2}}$, the only significant difference versus PRE was for $\bar{P}$_di,a_ after MIV, which was reduced at POST_1_ at 55 mmHg (−29%, *P* = 0.016, *d_z_* = 0.931). Owing to this reduction in $\bar{P}$_di,a_, diaphragm work was also reduced at this time point (*P* = 0.025, *d_z_* = 0.810). There were no effects of task or time on any of the other measures shown in Table 4. Overall, the findings point to a robust response of the diaphragm to hypercapnia, which was, for the most part, unaffected by respiratory loading.

**TABLE 4:** Pressure and ultrasound‐derived measures of diaphragm shortening at two levels of end‐tidal partial pressure of CO_2_ at baseline and at two time points after maximal isocapnic ventilation and inspiratory resistive loading.

	PRE		POST_1_		POST_2_	
Parameter	45 mmHg	55 mmHg	45 mHg	55 mmHg	45 mmHg	55 mHg
MIV						
$\bar{P}$_di,a_ (cmH_2_O)	8.5 (7.5)	13.9 (5.6)	5.8 (3.1)	9.8 (3.2)^*^	7.5 (4.5)	12.0 (5.2)
Excursion (cm)	3.58 (1.61)	4.84 (1.53)	3.39 (1.31)	4.72 (1.33)	3.55 (1.60)	5.14 (1.75)
Excursion time (s)	1.86 (0.60)	1.83 (0.43)	1.89 (0.50)	1.91 (0.46)	2.31 (0.74)	2.00 (0.48)
Excursion velocity (cm/s)	1.90 (0.54)	2.47 (1.23)	1.83 (0.63)	2.77 (1.30)	1.64 (0.90)	2.17 (1.60)
Work (cmH_2_O × cm)	37.7 (40.4)	69.9 (40.1)	22.4 (19.0)	47.2 (22.8)^*^	22.8 (9.9)	70.5 (46.2)
Power (cmH_2_O × cm/s)	19.2 (21.6)	38.9 (32.6)	11.4 (8.5)	29.5 (20.0)	10.6 (6.1)	28.1 (28.4)
IRL						
$\bar{P}$_di,a_ (cmH_2_O)	5.9 (4.1)	10.0 (4.1)	7.2 (3.4)	10.8 (3.8)	6.1 (3.0)	9.9 (3.0)
Excursion (cm)	3.08 (1.13)	4.50 (1.08)	3.43 (1.26)	4.87 (1.15)	2.59 (0.87)	4.47 (1.17)
Excursion time (s)	2.04 (0.68)	2.24 (0.91)	2.13 (0.60)	2.21 (1.58)	1.94 (0.58)	3.16 (1.96)
Excursion velocity (cm/s)	1.61 (0.66)	2.50 (0.89)	1.76 (0.94)	2.91 (1.22)	1.55 (0.88)	3.13 (1.60)
Work (cmH_2_O × cm)	21.3 (21.2)	45.7 (23.1)	26.5 (18.4)	52.7 (20.5)	16.9 (10.1)	44.9 (20.6)
Power (cmH_2_O × cm/s)	10.3 (9.9)	24.6 (11.8)	12.9 (8.1)	30.8 (14.2)	10.2 (8.9)	32.2 (20.9)

*Note*: Data are means (SD) for eight participants.

Pooled data for resting eupnoeic breathing at baseline (PRE): $\bar{P}$_di,a_ (6.8 ± 1.4 cmH_2_O), excursion (2.84 ± 1.50 cm), excursion time (1.81 ± 0.76 s), excursion velocity (1.55 ± 0.75 cm/s), work (23.6 ± 21.8 cmH_2_O × cm) and power (11.5 ± 11.5 cmH_2_O × cm/s).

Abbreviations: IRL, inspiratory resistive loading; MIV, maximal isocapnic ventilation; $\bar{P}$_di,a_, active component of mean transdiaphragmatic pressure.

* *P* < 0.05 versus PRE at same end‐tidal partial pressure of CO_2_.

## 4 DISCUSSION

### 4.1 Main findings

The aim of this study was to evaluate the feasibility of ultrasonography for the quantitative assessment of fatigue‐induced changes in contractile function of the human diaphragm. Using subcostal ultrasonography in combination with procedures that circumvent motivational bias, we assessed the shortening characteristics of the crural diaphragm before and after two distinct respiratory loading tasks. The main findings were threefold: (i) pressure loading (IRL) resulted in significant reductions in diaphragm power in response to supramaximal magnetic stimulation of the phrenic nerves, whereas flow loading (MIV) did not; (ii) the observed reductions in power were attributable primarily to decreases in velocity rather than force; and (iii) neither loading task affected the contractile responses of the diaphragm to progressive hypercapnia. Overall, the results provide partial support for our hypothesis that subcostal ultrasonography can be used to quantify changes in diaphragm contractile function attributable to fatigue.

### 4.2 Diaphragm contractile responses to phrenic nerve stimulation

Muscle fatigue is typically defined as a reversible decline in force or power resulting from contractile activity (Kent‐Braun et al., 2012). According to this definition, both MIV and IRL induced force‐related diaphragm fatigue, as evidenced by significant reductions, with large observed effects, in stimulation‐evoked twitch *P*_di_. Using similar protocols, Luo et al. (2001) observed significant reductions in twitch *P*_di_ following MIV and IRL in a small sample of healthy participants (*n* = 4 to 5). However, their reductions in twitch *P*_di_ (22% after MIV and 29% after IRL) were more pronounced than those observed in the present study (12% and 13%, respectively). Additionally, Luo et al. (2001) reported reductions of >10% in all participants, whereas in our study only three (MIV) and five (IRL) of eight participants exhibited such declines. These discrepancies might be attributable to differences in the training status of the participants. Although both studies recruited healthy volunteers, several participants in our study were competitive endurance athletes. Aerobic fitness has been suggested to offer some protection against exercise‐induced diaphragm fatigue (Babcock et al., 1996), which might explain the lower severity and prevalence of fatigue observed in our cohort. Additionally, the plateau ventilation during MIV in our study (72% of peak) was notably higher than the levels reported in previous studies by the same group (63%, Hamnegård et al., 1996; 65%, Mulvey et al., 1991). Whether aerobic fitness moderates diaphragm fatigue in conditions of maximal respiratory loading remains uncertain.

A novel feature of the present study was the use of subcostal ultrasonography to quantify the contractile shortening of the crural diaphragm. In the resting unfatigued state, craniocaudal excursion during phrenic stimulation (1.6 cm) was similar to values reported previously for a maximal sniff, which ranged from 1.5 (Scott et al., 2006) to 3.0 cm (Henke et al., 2019). This similarity is likely to reflect the comparable levels of muscle shortening during quasi‐isometric contractions. Indeed, studies in dogs have demonstrated substantial crural shortening during both supramaximal phrenic nerve stimulation and inspiratory efforts against occlusion (Fitting et al., 1985; Newman et al., 1984). Conversely, crural velocity in our study (14.5 cm/s) exceeded previously reported values for a maximal sniff, which ranged from 6.4 (Spiesshoefer et al., 2019) to 12 cm/s (Boussuges et al., 2021). Given that crural velocity is a function of craniocaudal excursion and contraction time (velocity = excursion/time), the higher velocity during phrenic stimulation implies a shorter contraction time. Ultrasound‐derived excursion time in our study (0.12 s) closely matched the previously reported time to achieve peak twitch *P*_di_ in healthy humans (0.08 s; Bellemare et al., 1986). However, our excursion time was notably shorter than the values for a maximal sniff, which ranged from 0.36 (Spiesshoefer et al., 2019) to 0.3 s (Boussuges et al., 2021). This difference is likely to arise from the isolated action of the diaphragm during twitch contractions compared with the coordinated action of multiple inspiratory muscles during a sniff. Other factors, such as participant demographics (e.g., sex, age, stature and mass), body position (e.g., upright vs. supine vs. semi‐recumbent) and analysis procedure (e.g., conventional vs. anatomical M‐Mode), are also likely to account for some of the observed differences across studies.

Our data show that IRL led to significant reductions, with large observed effects, in ultrasound‐derived measures of crural shortening. Specifically, a reduction in crural excursion was observed at the initial post‐task time point. This reduction, coupled with small, non‐significant increases in excursion time, resulted in significant decreases in mean excursion velocity at both post‐task time points. The reduction in excursion, alongside the concurrent decline in twitch *P*_di_ at the same time point, resulted in a significant reduction in diaphragm work (work = force × distance). Likewise, the combined effects of IRL on twitch *P*_di_ and excursion velocity resulted in significant reductions in the mechanical power of the diaphragm (power = force × velocity). Notably, these reductions in power were driven primarily by decreases in velocity, with reductions in force playing only a secondary role. Unexpectedly, MIV had no significant effect on any of the ultrasound‐derived measures. Diaphragm force output was significantly lower during MIV compared with IRL, and the diaphragm pressure–time index during MIV (0.16) was only slightly above the critical threshold for diaphragm fatigue (0.15–0.18; Bellemare & Grassino, 1982). Furthermore, the ratio of $\bar{P}$_di_ to $\bar{P}$_oe_ was significantly higher during MIV, suggesting that the diaphragm contributed less to overall pressure generation than other inspiratory muscles. Thus, the observed differences in crural shortening between the two tasks might be explained by variations in both absolute and relative loading.

The observation that IRL, but not MIV, induced significant reductions in ultrasound‐derived measures of crural shortening seems to be inconsistent with the principle of task specificity, which posits that fatigue is determined by the specific demands of the task (Enoka & Stuart, 1992). The two loading tasks were chosen on the basis that they represent opposite ends of the force–velocity spectrum, with IRL characterized by high force and MIV by high velocity. However, it is important to emphasize that these characteristics cannot be entirely isolated in vivo. For instance, IRL requires dynamic muscle contraction, with rapid force development during the ‘rising edge’ of the square‐wave increase in pressure, before transitioning to the sustained isometric phase. Conversely, MIV, while emphasizing high shortening velocities, also demands substantial pressure generation (Table 2). Thus, the observed differences in fatigue cannot be attributable solely to force or velocity; other factors, such as loading duration, contraction frequency, duty cycle and muscle recruitment, are also likely to play significant roles. Given the complexity and interrelated nature of these interactions, we refrained from direct statistical comparisons. This decision reflects the desire to avoid oversimplifying the intricate interplay of factors that contribute to diaphragm fatigue in different loading conditions.

Although we are the first to use ultrasonography to assess contractile fatigue of the human diaphragm, previous studies have used alternative methods to investigate this phenomenon. For instance, McCool et al. (1992) assessed the maximal pressure–flow capacity of the inspiratory muscles in response to fatigue induced via a force/pressure task (IRL with a target *P*_oe_) or a velocity/flow task (eucapnic hyperpnoea with a target peak inspiratory flow). Their results showed a significantly greater decline in maximal inspiratory muscle pressure (*P*_oe_) after the force/pressure task compared with the velocity/flow task (−25% vs. −8%). In contrast, maximal *P*_di_ and maximal inspiratory flow decreased more after the velocity/flow task than after the force/pressure task (−16% vs. −3%). More recently, Sarmento et al. (2021) and Lima et al. (2022) used optoelectronic plethysmography to examine the effects of inspiratory pressure‐threshold loading on diaphragm shortening; both studies reported reductions in the shortening velocity and mechanical power of the inspiratory ribcage muscles, but not of the diaphragm. A limitation of all three studies is that contractile function was assessed indirectly using maximal inspiratory manoeuvres, which, as noted earlier (Section 1), are highly dependent on participant effort and motivation. An additional concern is the potential inaccuracy of airflow measured at the mouth as an index of diaphragm shortening velocity (McCool et al., 1992). In this regard, Newman et al. (1984) found no clear relationship between instantaneous mouth flow and directly measured crural shortening velocity in dogs. Likewise, our recent work in healthy humans demonstrated considerable variability in the relationship between mean inspiratory flow and ultrasound‐derived crural velocity (Illidi & Romer, 2022). In summary, the combined approach of using objective, effort‐independent techniques alongside direct visualization of the diaphragm provides a more accurate assessment of diaphragm contractile function and fatigue than the methods used in previous studies.

The in vivo nature of the present study limits our ability to pinpoint the precise mechanisms for the observed reductions in diaphragm pressure and crural shortening. Fatigue‐related declines in contractile function in response to low‐frequency stimulation (e.g., ‘twitch’ contractions) have been linked to disruptions in excitation–contraction coupling and/or structural damage to contractile proteins (Kent‐Braun et al., 2012). Cellular and molecular research suggests that such impairments might result from the accumulation of intracellular metabolites consequent to increased anaerobic metabolism (Sundberg & Fitts, 2019). Elevated intracellular H^+^ concentrations, for example, have been shown to inhibit cross‐bridge force, velocity and power, while also reducing myofilament sensitivity to Ca^2+^. Likewise, increased *P*_i_ concentrations have been shown to reduce cross‐bridge force and power, in addition to Ca^2+^ availability and sensitivity. When both H^+^ and *P*_i_ are elevated, the combined effects on muscle power output are synergistic, potentially owing to alterations in the binding affinities of the myosin–actin interaction during the cross‐bridge cycle. The extent to which other compounds contribute to loading‐induced impairments in contractile function is not entirely clear. In our study, contractile function did not recover fully within 30 min after loading, indicating the potential involvement of compounds that are capable of causing more long‐lasting structural damage, such as reactive O_2_ and N_2_ species (Reid, 2016). These compounds are known to interact with cellular proteins, leading to oxidative damage that might impair muscle recovery and prolong the effects of fatigue. Further research is needed to clarify the contribution of these molecular factors to fatigue‐related reductions in diaphragm contractile function.

### 4.3 Diaphragm contractile responses to progressive hypercapnia

To assess the influence of respiratory loading on the shortening of the crural diaphragm during dynamic tidal breathing, we needed to establish a broad and consistent range of ventilatory responses. To this end, we used a modified version of Read's CO_2_ rebreathing protocol (Read, 1967), during which participants breathed from a bag filled with 5% CO_2_ in O_2_ rather than the standard 7% CO_2_. This modification was expected to slow the rate of CO_2_ accumulation, thereby prolonging the duration of the rebreathing trials and facilitating more extensive data collection. Although the protocol elicited a robust ventilatory response in most participants, there was considerable variability between individuals (0.28–5.50 L/min/mmHg). This variability is consistent with previous research. For example, Jensen et al. (2010) reported between‐participant CV values ranging from 26% to 77% for the overall ventilatory response to a modified hypercapnic–hyperoxic rebreathing procedure. Furthermore, the mean increase in ventilation in our study (2.5‐fold) was less than the increases typically reported for modified rebreathing procedures (Jensen et al., 2010). This lesser response is probably attributable to the lower $P_{\mathrm{ET},CO_{2}}$ cut‐off used in our study (55 mmHg) compared with the higher threshold used in previous research (≥60 mmHg).

Contrary to our hypothesis, respiratory loading did not influence the crural shortening responses to CO_2_ rebreathing. This finding could be attributable to high within‐day variability in the ventilatory response to CO_2_. Previous studies have reported substantial within‐day, between‐occasion variability for the overall ventilatory response to a modified hypercapnic–hyperoxic rebreathing procedure, with CV values for the slope of ventilation versus $P_{\mathrm{ET},CO_{2}}$ ranging from 18% (Sahn et al., 1977) to 26% (Jensen et al., 2010). Such variability might also explain inconsistencies in the literature regarding the effects of prior respiratory loading and fatigue on subsequent ventilatory and pressure responses to CO_2_. For instance, Mador and Tobin (1992) reported a modest but significant reduction in the ventilatory response slope following global inspiratory muscle fatigue (IRL, 80% PI_max_), but no significant changes in ventilation at specific CO_2_ levels and no evidence of rapid, shallow breathing. In contrast, Yan, Lichros et al. (1993), using an identical loading protocol, found that global inspiratory muscle fatigue reduced tidal volume and increased respiratory frequency at discrete $P_{\mathrm{ET},CO_{2}}$ levels, but did not affect minute ventilation, duty cycle or mean inspiratory flow. When loading was applied specifically to the diaphragm (IRL, 60% *P*_di,max_), Yan, Sliwinski et al. (1993) found no significant effects on ventilatory or breathing pattern responses at discrete $P_{\mathrm{ET},CO_{2}}$ levels, although they did observe, in line with the present study, a significant reduction in tidal *P*_di_. A reduction in tidal *P*_di_ implies a shift towards an increased reliance on the inspiratory ribcage muscles over the diaphragm during inspiration. However, a more recent study showed that diaphragm fatigue induced using loading protocols similar to those in the present study did not affect neural drive to the crural diaphragm (Luo et al., 2001). This finding suggests that respiratory loading does not alter diaphragm recruitment. In summary, the high variability in ventilatory and pressure responses to CO_2_ might account for the mixed findings regarding the effects of respiratory loading on subsequent ventilatory, pressure and shortening responses to CO_2_.

To our knowledge, only one other study has investigated the influence of respiratory loading on diaphragm contractile responses to reflexively driven increases in tidal breathing. Kocis et al. (1997), using a piglet model of fatigue, reported a significant reduction in diaphragm force (*P*_di_ −25%) and an even greater reduction in excursion velocity (−38%) during CO_2_‐induced hyperpnoea after fatigue was induced via repetitive electrical stimulation of the phrenic nerves. The discrepancy between the findings of Kocis et al. (1997) and our study might be attributable to differences in motor unit recruitment. Electrical stimulation of motor nerves elicits non‐selective recruitment of motor units, including the fatigue‐prone type IIx fibres, whereas voluntary muscle activation typically prioritizes the recruitment of the more fatigue‐resistant type I fibres (Bickel et al., 2011). This difference in recruitment pattern might account for the greater fatigue observed by Kocis et al. (1997).

### 4.4 Technical considerations

To draw valid conclusions about fatigue, it is essential that phrenic nerve stimulation is maximal. All participants exhibited a plateau in twitch *P*_di_ with increasing stimulation intensity before each task (Figure 4), thereby confirming maximal stimulation. Although we did not assess crural shortening during the incremental stimulation protocol, we infer that plateaus did occur based on the assumption that the diaphragm adheres to the same force–velocity relationship as do other skeletal muscles (Pengelly et al., 1971). We also assume that nerve stimulation was maximal both before and after loading. Owing to logistical constraints, we were unable to measure crural EMG directly and therefore cannot state with absolute certainty that the neural stimulus was maximal for the duration of each trial. However, all stimulations were delivered at 100% of stimulator output, and the coil positions were carefully marked at baseline to ensure precise repositioning after loading. Additionally, lung volume (diaphragm fibre length) and abdominal compliance were controlled during stimulation by monitoring end‐expiratory oesophageal and gastric pressures.

Our loading protocols were similar to those previously shown to induce significant, long‐lasting reductions in twitch *P*_di_ (Luo et al., 2001). To control for potentiation effects (Mador et al., 1994), we were careful to standardize the timing of post‐load twitches (i.e., 10 min) and the order of the experimental conditions (i.e., phrenic stimulation before CO_2_ rebreathing). Despite these precautions, the persistence of some potentiation, especially after high‐force IRL contractions, is a valid concern. Notably, the observed reductions in twitch *P*_di_ at 30 min were similar to or greater than those at 10 min. Given that fatigue and potentiation can co‐exist (Rassier & MacIntosh, 2000), it is possible that we might have underestimated the magnitude of force fatigue. Interestingly, there is some evidence that prior muscular activity might also increase muscle fibre shortening. For instance, MacIntosh and Bryan (2002) reported a 51% increase in the shortening velocity of rat gastrocnemius fibres with stimulation‐evoked tetanic contractions. Given that the human diaphragm has a higher proportion of slow‐twitch fibres than the rat gastrocnemius, it is reasonable to suppose that the potentiation effects might have been less pronounced in the present study. Indeed, our own (unpublished) observations in healthy participants (*n* = 10) show a modest, albeit non‐significant, increase in crural velocity (11%), alongside a much larger, significant increase in twitch *P*_di_ (25%), in response to maximal static inspiratory efforts.

A 10–15 min delay between respiratory loading and the initial post‐load measurements was implemented to allow breathing to return to normal and to minimize the effects of postactivation potentiation. Fatigue resulting from low‐frequency stimulation is considered ‘long‐lasting’ (Travaline et al., 1997). As such, the recovery period probably did not significantly influence the magnitude of force‐related fatigue (i.e., twitch *P*_di_). However, evidence from animal studies suggests that the velocity properties of muscle recover more rapidly. For instance, Hatcher and Luff (1987) demonstrated that the maximal shortening velocity of fast‐twitch fibres recovered more rapidly than maximal isometric tension following fatiguing repetitive isometric stimulation in anaesthetized cats. Such rapid recovery of velocity suggests that by the time our post‐load measurements were taken, some recovery of velocity (and power) might already have occurred, resulting in an underestimation of fatigue.

A further consideration is the potential influence of interaction and carry‐over effects. Previous research has shown that elevated CO_2_ above normocapnic levels can increase the ventilatory response to a subsequent challenge. However, this effect was observed only after prolonged CO_2_ exposure (Griffin et al., 2012). Moreover, previous work has reported non‐systematic differences in the ventilatory response to repeated CO_2_ rebreathing trials over periods of 2–6 h (Jensen et al., 2010; Sahn et al., 1977). Therefore, it seems highly unlikely that the repeated, short‐term hypercapnia in our study significantly influenced the ventilatory response to subsequent rebreathing trials. Another factor to consider is the potential impact of hypercapnic acidosis on diaphragm function. Moderate hypercapnia (15 min, 7.5% CO_2_) has been shown to reduce maximal voluntary *P*_di_ in a small sample of participants (Juan et al., 1984). However, larger studies have found no significant effects of hypercapnia (6–20 min, 7–8% CO_2_) on twitch *P*_di_ (Mador et al., 1997; Wan et al., 2018). Acute hypercapnia during 2 min of fatiguing MIV has been shown to exacerbate reductions in twitch *P*_di_ immediately after MIV, but this effect reversed rapidly with the return to normocapnia (Rafferty et al., 1999). Although the impact of hypercapnic acidosis on crural shortening remains unknown, we controlled for any potential influences by performing evoked twitches before CO_2_ rebreathing trials and allowing for adequate recovery between trials. Thus, we think it is highly unlikely that the moderate, short‐term hypercapnia used in the present study had a significant effect on contractile function.

A strength of the present study is the use of subcostal ultrasonography to quantify fatigue‐induced changes in crural shortening. This technique permits real‐time, non‐invasive visualization of diaphragm contraction. To minimize variability and ensure data consistency, all ultrasound data were acquired and analysed by the same investigator (C.R.I.) in accordance with established guidelines (Laursen et al., 2021; Orde et al., 2016). We focused on the right hemidiaphragm because the anatomical location of the liver provides an acoustic window that enhances the transmission of ultrasound waves. Although we did not measure the motion of the left hemidiaphragm, evidence in dogs suggests a symmetrical movement pattern during phrenic stimulation and spontaneous breathing (De Troyer et al., 2003; Newman et al., 1984). A further assumption is that our measures of crural shortening during involuntary contractions are representative of the entire diaphragm. Studies in anaesthetized dogs have found no significant differences in muscle shortening and velocity between the costal and crural segments during spontaneous breathing, phrenic stimulation or progressive hypercapnia (Fitting et al., 1985; Newman et al., 1984). Studies in unanaesthetized dogs have also noted comparable levels of shortening for the costal and crural segments during progressive hypoxia or hypercapnia (Easton et al., 1995, 1993). In contrast, a more recent study involving a larger sample of unanaesthetized dogs found significantly less shortening of the crural segment during progressive hypercapnia (Tagliabue et al., 2019). Together, these findings suggest that the two segments have distinct, yet interacting mechanical roles.

### 4.5 Perspectives and future directions

This study highlights the feasibility and utility of combining subcostal ultrasonography with phrenic nerve stimulation to assess contractile fatigue of the human diaphragm. Although our focus was on evaluating diaphragm fatigue in response to external loading, this method is also well suited for other contexts in which the inspiratory muscles are subjected to increased loads. For instance, the method could offer valuable insights into diaphragm contractile responses to dynamic, whole‐body exercise. It is well established that intense, sustained exercise can induce substantial force‐related fatigue, as evidenced by significant reductions in stimulation‐evoked *P*_di_ (Johnson et al., 1993). Moreover, during exercise, the diaphragm acts primarily as a ‘flow generator’, with its mechanical power mainly a function of shortening velocity rather than force output (Aliverti et al., 1997). Thus, our approach to quantifying crural shortening could provide a more comprehensive characterization of exercise‐induced changes in diaphragm contractile function, extending beyond conventional measures of force/pressure.

Furthermore, coupling subcostal ultrasonography with phrenic stimulation could yield valuable insights into diaphragm dysfunction and its contribution to functional impairments in healthy older adults and patients with inspiratory muscle weakness. The method also holds promise for evaluating interventions aimed at enhancing diaphragm function. Both pressure and flow loading have been used in athletic and rehabilitation settings to improve respiratory muscle function (Illidi et al., 2023). Accurate assessment of diaphragm contractile properties using the method outlined herein could help to inform the development of more targeted and effective training regimens.

## 5 CONCLUSION

Synchronous recording of pressure data and ultrasound traces during phrenic nerve stimulation has proved to be a feasible and effective method for quantifying changes in diaphragm contractile function owing to fatigue. This innovative approach has the potential to provide valuable insights into the contractile properties of this essential muscle across a wide range of applications and population subgroups. Furthermore, it offers considerable promise for informing the development of targeted interventions aimed at enhancing inspiratory muscle function in health and disease.

## AUTHOR CONTRIBUTIONS

The experiments were conducted at Brunel University London. Lee Romer and Camilla Illidi conceived and designed the study, and both were involved in data collection, analysis and interpretation. Lee Romer and Camilla Illidi drafted the manuscript and revised it critically for important intellectual content. Lee Romer and Camilla Illidi approved the final version of the manuscript and agree to be accountable for all aspects of the work in ensuring that questions related to the accuracy or integrity of any part of the work are appropriately investigated and resolved. Both persons designated as authors qualify for authorship, and all those who qualify for authorship are listed.

## CONFLICT OF INTEREST

None declared.

## FUNDING INFORMATION

None.

## Data Availability

The data that support the findings of this study are available from the corresponding author upon reasonable request.
